# Risk factor control, adherence to medication and follow up visit, five years after coronary artery bypass graft surgery

**DOI:** 10.15171/jcvtr.2016.31

**Published:** 2016-12-30

**Authors:** Arsalan Salari, Tolou Hasandokht, Marjan Mahdavi-Roshan, Jalal Kheirkhah, Mahboueh Gholipour, Mahsa Pouradollah Tootkaoni

**Affiliations:** ^1^Guilan Interventional Cardiovascular Research Center, Heshmat Hospital, Guilan University of Medical Sciences, Rasht, Iran; ^2^Community Medicine Department, Faculty Of Medicine, Guilan University of Medical Sciences, Rasht, Iran

**Keywords:** Medication Adherence, Risk Factor Control, Smoking, Coronary Artery Bypass Graft

## Abstract

***Introduction:*** Inadequate adherence to medication and follow up visits were proposed correlated with cardiovascular mortality and complications. This study was planned to evaluate medication and follow up adherence and risk factor control in patients with coronary artery disease 5 years after coronary artery bypass grafting (CABG).

***Methods:*** In this retrospective cohort study, adult patients who underwent CABG in 2010 were enrolled. Conventional and probable risk factor control and adherence to medication and follow up visits were assessed.

***Results:*** 196 patients were recruited to the study. Uncontrolled blood pressure, blood glucose and low-density lipoprotein (LDL)were reported in 48%, 61% and 32% of patients, respectively. More than 63% of former smokers restarted smoking during 6-12 months after bypass. Poor medication adherence was present in 10.7% in the study population. The last follow up visit time for 30% of patients was later than 12 months after CABG.

***Conclusion:*** Poor risk factors control and adherence to follow up visits was common among patients undergoing CABG.

## Introduction

Coronary artery bypass graft (CABG) was known as an important and common method for coronary revascularization in patients with coronary artery disease (CAD). After CABG, recurrent coronary events have been frequently reported as a result of atherosclerotic process.^1^ Risk factor management with medication accompanied with lifestyle intervention, including salt reduction, weight loss, cigarette smoking cession and regular physical activity reduce recurrent cardiovascular event in CAD patients.^1-4^ Cardiovascular guidelines recommend medical therapy for patients with CAD to decrease cardiovascular events as a secondary prevention.^5,6^ However, evidence showed that achieving risk factors target level in real clinical settings is difficult.^7,8^ Secondary prevention and risk factor control in patients undergoing revascularization procedure such as CABG or percutaneous intervention has been reported were poor and unsatisfactory.^8,9^ A recent meta-analysis concluded there are low adherence to primary and secondary prevention medication in healthy people and patients with a myocardial infarction history, respectively.^5^ Evidence showed poor medication adherence were associated with cardiovascular mortality, cardiovascular rehospitalizations and revascularization procedures.^6,10^ Guidelines from the American Diabetes Association, Adult Treatment Panel III, National Committee on Prevention, Detection, Evaluation, and Treatment of High Blood Pressure and American College of Cardiology/American Heart Association were specified the goal of each risk factors such as hypertension, diabetes, dyslipidemia and tobacco smoking. We examined the medication and follow up visit adherence and risk factor control level in CAD patients 5 years after CABG.

## Materials and Methods

### Design

In a retrospective cohort study, adult patient were hospitalized for CABG were identiﬁed from hospital discharge lists in 2010 in a teaching hospital of the northern part of Iran. All included patients were discharged alive from the hospital. Design and protocol of the study approved and fund by Vice-Chancellor for Research of Guilan University of medical science and Interventional Cardiovascular Research Center (ICRC). According to discharge list, in a total of 254 patients were called and invited to the clinic for study by a trained nurse. A specific date and time was determined for each patient. Finally 196 patients were recruited in the study, since 15 were dead, 32 for wrong registered cell number, 4 for home transition and 7 rejected to involve the study for long distance to clinic.

### Patient characteristics

Demographic, underlying disease and risk factors included age, sex, living situation (urban or rural), diabetes, hypertension, dyslipidemia, smoking, new opium consumption and dietary status. New opium user defined as those patients use opium after CABG as due to preventive effect of opium. Medical history was defined based on patient report or medications. Anthropometric parameters like height, weight were measured in light clothes without shoes by a precalibrated digital SECA scale and portable stadiometer. Blood pressure (BP) was measured with a calibrated Omron M7 sphygmomanometer (HEM-780-E) in sitting position after 15 minutes rest by a trained nurse. All data were collected by a trained nurse on the predetermined date for visit.

Laboratory data, such as low-density lipoprotein (LDL), fasting blood sugar (FBS) and serum 25-OHD were derived from last patient laboratory data during prior 3 months. For those patients who did not have documented laboratory data, we examined fasting blood test.

Uncontrolled risk factors were defined as (1) HbA1c > 7,^11^ (2) BP >140/90 mm Hg, BP >130/80 mm Hg in diabetes people,^12^ (3) LDL > 100 mg/dL for high-risk groups,^13^ (4) continued smoking and (5) obesity (body mass index (BMI) ≥30 kg/m^2^).^8^

According to US Endocrine Society guideline,^14^ vitamin D deficiency defines as 25-OHD levels less than 20 ng/mL and vitamin D insufficiency defines as 25-OHD levels between 21 and 29 ng/mL.

Smoking status were divided to two category (1) former smoker: who smoked cigarettes before CABG, (2) current smoker: who continued cigarettes smoking after CABG even one cigarette/d. Duration of smoking cessation was assessed by 2 questions. (1) Do you stop smoking after CABG? Possible answer was yes or no. (2) When did you start smoking again? Patient’s reply was (*a*) less than 6 months, (*b*) 6-12 months, and (*c*) more than 12 months. All patients were asked about opium consumption as orally or smoking. Furthermore, the time of opium usage were asked. Participants’ answer included (*a*) before surgery and (*b*) after surgery.

Overall medication adherence was assessed by one question.^14^ “Do you take your medications as the doctor prescribed?” Possible responses were: (1) all of the time, (2) most of the time, and (3) usually irregular/discontinued.

We assessed the frequency of follow up visits using the 1 question. (1) What is the last your doctor visit? Patient’s answer was presented as (*a*) during 6 months ago, (*b*) between 6-12 months, (*c*) more than 12 months ago. All patients were asked another question” who was your doctor in the last visit? Possible answers were (*a*) cardiologist, (*b*) general physician.

### Dietary assessment

Assessment of dietary intake was undertaken with Semi-quantitative Food Frequency Questionnaire that has reasonable relative validity and reliability for nutrient intakes which used in the previous studies.^15 ,16^ To score the dietary diversity, based on the Food Guide Pyramid, five food groups of bread and grains, fruits, vegetables, meats and their substitutions and dairy foods were used. According to Haines et al^17^ the main five groups were divided into twenty three subgroups. To be counted as a consumer for any of the food group categories, a respondent needed to consume at least one-half serving per day. Each of the five broad food categories receives a maximum diversity score of two out of the ten possible score points. For calculation of the score of each group, we divided the number of subgroups consumed by the total number of subgroups of each main group and then we multiplied this by two. For example, if a person consumed at least one-half serving from two of the possible meat categories, he or she would receive a subgroup score of (2/4) × 2 =1. Total dietary diversity (DD) score was the sum of the scores of the five food groups. The higher DD score was shown the greater variety. Similar to Mirmiran et al study,^16^ we divided the study population regard to DD score into two group (less than 6 and greater than 6).

### Statistical analysis

The SPSS (Windows version 11, SPSS Inc., Chicago, USA) was used for data analysis. The chi-square test and independent *t* test were used to assess the qualitative or numerical variables, respectively. A *P* value of ≤0.05 was used as the criterion of statistical signiﬁcance.

## Results

Baseline characteristics of the study population are presented in Table 1. The mean age of the population was 57 (8.1) years. The proportion of male gender was 67%. Women were at a significantly higher mean age than men (63.1 versus 51.1 years; *P* = 0.01). Frequency of diabetes, hypertension and hypercholesterolemia in all study cohorts was 43%, 52% and 32%, respectively. The proportion of smoking and opium consumption was 9.6% in all population. Majority of participant (89%) use opium orally and 84% of them start opium consumption after surgery. The mean score of total DD was 3.9 ± 1.06 (range: 0.7-7.2) for all study population. The maximum and minimum scores for diet diversity, respectively were related to fruit (0.94 ± 0.33) and meat (0.32 ± 0.12) groups.

**Table 1:** Baseline characteristics of the study sample

	** Total (196) **	**Men (112, 67%)**	**Women (84, 32%)**	***P***
Age (mean + SD, years)	57 (8.1)	51.1 (8.6)	63.1 (7.7)	0.01
Living situation (n, %)				0.7
Urban	154 (79.2)	85 (76.9)	69 (82)	
Rural	42 (20.8)	27 (23.1)	15 (17)	
Education (n, %)				0.002
<12 years	64 (32)	25 (22)	39 (46)	
≥Diploma	132 (67)	87 (77)	45 (53)	
Diabetes (n, %)	86 (43)	40 (35)	46 (54)	0.02
Hypertension (n, %)	102 (52)	58 (51)	44 (52)	0.09
Hypercholesterolemia (n, %)	58 (32)	38 (33)	20 (23)	0.06
Body mass index (n, %)				0.001
≤25	114 (58)	74 (66)	40 (47)	
Overweight & Obesity	82 (42)	38 (33)	44 (52)	
Blood pressure (mean + SD)				
Systolic BP	156 (8.7)	167 (9.1)	145 (6.2)	0.02
Diastolic BP	89 (3.1)	93 (6.3)	85 (2.3)	0.03
HbA1C, mean (SD)	8.4 (2.1)	7.9 (3.1)	8.9 (2.3)	0.02
LDL (mean + SD)	109 (13)	111 (12)	108 (9)	0.3
Vitamin D (mean + SD)	11 (4.1)	11 (3.1)	7 (2)	0.08
Vitamin D deficiency (n, %)	131 (66)	67 (60)	64 (76)	0.2
Vitamin D insufficiency (n, %)	60 (30)	30 (26)	30 (35)	0.6
Smoker-former (n, %)	55 (28)	53 (47)	2 (2)	0.000
Opium user (n, %)	19 (9.6)	11 (9.8)	8 (9.5)	0. 1
Dietary diversity score < 6 (n, %)	134 (68.3)	82 (73.2)	52 (61.9)	0.4
Dietary diversity score (mean ± SD)	3.3 ±1.06	3.4±0.9	3.2±1.1	0.5
Bread & grains (mean ± SD)	084±0.3	0.86±0.3	0.82±0.2	0.5
Meat (mean ± SD)	032±0.3	0.33±0.3	0.30±0.4	0.7
Fruit (mean ± SD)	0.94±0.3	0.93±0.2	0.96±0.4	0.6
Vegetables (mean ± SD)	0.41±0.4	0.42±0.4	0.39±0.3	0.7
Dairy (mean ± SD)	0.83±0.5	0.86±0.6	0.77±0.5	0.5

Risk factor control and medication adherence category were shown in Table 2. A total of 28% of patients reported taking their medicine all of the times while, 10.7% of them reported taking medications usually irregular or discontinued. Among the hypertensive group, 48% did not achieve the treated goal. The frequency of uncontrolled hypertension and hypercholesterolemia were statistically significantly greater in patients with irregular use of medicine (46% and 58% respectively). More than 60% of patients with DM did not achieve the treatment goal. The proportion of uncontrolled diabetes was not higher in patients who reports irregular taking medication (0.09) (Table 2).

**Table 2:** Risk factors control according to Medication adherence

	**Total** ** (196) **	**All of the time** **(56, 28.5%)**	**Most of the time** **(119, 60.7%)**	**Usually irregular/ discontinued** **(21, 10.7%)**	***P*** ^d^
Overall hypertension, n (%)	102 (52)				
Hypertension-treated not at goal^a^	49 (48)	10 (20)	16 (32)	23 (46)	0.001
Non diabetic	29 (59)	7 (24)	10 (34)	12 (41)	0.02
diabetic	20 (40)	3 (15)	6 (30)	11 (55)	0.07
Overall hyperlipidemia, n (%)	58 (32)				
Hypercholesterolemia-treated not at goal^b^	31 (53)	4 (12)	9 (29)	18 (58)	0.03
Overall diabetes, n (%)	86 (43)				
Diabetes-treated not at goal^c^	53 (61)	20 (37)	17 (32)	16 (30)	0.09

^a^BP >140/90 mm Hg, BP >130/80 mm Hg in diabetes people. ^b^ LDL > 100 mg/dL. ^c^Fasting blood sugar >126 mg/dL. ^d^Chi-square test.

As shown in Table 3, the proportion of uncontrolled hypertension, hypercholesterolemia, diabetes and overweight were statistically significant different based on the last follow up visit time (*P* < 0.05). The last follow up visit time in the majority of these patients was more than 12 months ago. A total of 9% of patients continually smoke after CABG. The last follow up visit time for more than 52% of current smoker were greater than 12 months. Diet diversity score in 66% of study populations was lower than 5. There was no statistically significant difference in the frequency of patients with low DDS regard to frequency of follow up visits (*P* > 0.05).

**Table 3:** Risk factors control according to last follow up visit

	**Total** ** (196) **	**<6 month ago,** **42 (21%)**	**6-12 months,** **94 (32%)**	**>12 months,** **60 (30%)**	***P*** ^a^
Hypertension-treated not at goal, n (%)	49 (48)				
Non-diabetic	29 (59)	8 (15)	16 (31)	27 (52)	0.001
Diabetic	20 (40)	7 (24)	11 (37)	11 (37)	0.1
Hypercholesterolemia -treated not at goal, n (%)	31 (53)	5 (12)	7 (14)	29 (70)	0.01
Diabetes-treated not at goal, n (%)	53 (61)	12 (22)	19 (35)	22 (41)	0.04
Smoker-former, n (%)	55 (28)				
Smoker-current	19 (9)	3 (15)	6 (31)	10 (52)	0.01
BMI >30 kg/m^2^	32 (16)	8 (25)	8 (25)	16 (50)	0.01
Dietary diversity score < 6, n (%)	134 (68.3)	30 (71.4)	61 (64.8)	43 (71.6)	0.3

^a^Chi-square test.

Only 21% of study patients see their doctor in recent 6 months and 82% of them lived in a rural area. Interestingly, the majority of rural patients (32/34) received medications and health education plan from family physicians in rural health care centers.

Approximately 34% (19/55) of the former smoker started smoking again and noteworthy, 63% of current smoker started smoking during 6-12 months after bypass. Only, 2 people continued smoking following surgery and another 26% of current smoker started again after 1 year.

## Discussion

This study found more than 50% of patients take medicine regularly, but only 21% have acceptable follow up adherence. More than 50% of major risk factors like DM, hypertension and hypercholesterolemia did not achieve therapeutic target. And also, the proportion of uncontrolled risk factors dramatically increased with irregular consumption of medication and non adherence to follow up visits. Parallel with our study, the medication adherence was reported in 55% of patients 6–24 months after CABG.^18^ In line with the present study, Krousel-Wood et al in a review of literature discussed about the importance of patients’ adherence to antihypertensive medicine in BP control.^19^ Finding from a study on 137277 patients showed better medication adherence was significantly associated with lower rates of rehospitalizations in diabetic and hypertensive cases and also reduce cost of disease management.^20^

This study showed that uncontrolled DM did not associate with medication adherence, but related to commitment to follow up visits. Recent review showed a positive effect of adherence to anti-diabetes medicines on blood glucose control.^21^ However, similar association did not report among low income populations probably due to different race/ethnicity.^21^ Our finding may be explained by this fact that medical therapy without lifestyle modification in diabetes treatment is insufficient.^22^ Frequent receiving healthy lifestyle recommendation like more exercise and eat healthy food due to adherence to follow up visits improve the serum glucose level. In this study, more cases with uncontrolled diabetes take medication regularly. On the other hand, low percentage of them recently sees their doctor. The continuing use of inappropriate dose of anti-diabetes medicine due to poor adherence to follow up visits may explain this finding.

The notable finding of our study was the high proportions of patients with good adherent to follow up visits receive their treatment from a family physician in rural health care centers. Being accessible and inexpensive of Iranian rural health care services (the Behvarz system) provides better patient management. As, Farzadfar et al in a large data study showed the effectiveness of rural primary health care system on the non communicable disease control.^23^

Opium consumption was reported in approximately 10% of patients. Considerably, the majority of them start an opium after bypass surgery for probable beneficial consequences of opium. Parallel to our study, in Kerman coronary artery disease risk factors study, 17.8% of men and 3.0% of women were opium user.^24^ In spite of general population belief, several studies revealed the harmful effect of opium on cardiovascular risk factors^25-27^ and severity of coronary atherosclerosis.^28^

The successful smoking cession rate in the present study was 65%. Similarly, in Kadda et al study,^29^ 83% of patients stopped smoking during 1 year following CABG. The present study showed that most current smoker started smoking through 6-12 month after surgery. Moreover, more than 50% of current smokers did not see their doctors during recent year. Hence, performing regular and precise visit programs with quit smoking emphasis seems to be effective for successful smoking cessation. Ischemic pain and symptoms decreasing as well as physical function improvement may be due to restart cigarette smoking. This is in line with other studies^30^ indicated a high numbers of smokers admitted to hospital after myocardial infarction or cardiac surgery, start smoking again within a year. Smokers with cardiovascular disease should undergo regular intensive cessation behavioral intervention after hospital discharge.^31,32^

In the present study, more than half of patients obtained poor diet diversity scores and it was not related to follow up visits frequency. In a study on Tehranian adults the mean DDS was higher than the mean score in our study.^15^ The probable reason for poor DDS in the present study was; the lower economic status of our patients compared with Tehranian adult. Culturally, in our study area, patients with higher economic state receive their health services from private hospitals. While, we studied patients underwent CABG in a public hospital can lead to poor diet score. The association of better atherosclerotic risk factors controls with a higher diversity diet score in Azadbakht study investigated.^15^ In other words, a signiﬁcant association between food diversity and arterial wall thickness index were reported.^33^

We could not assess the information of 22% of patients undergoing surgery, due to wrong registered call number or home transition can somewhat limit our result. However, an important aspect of the current study refers to the high prevalence of opium consumption among the study population with therapeutic goal. Health education to change people’s perception seems to be required.

In conclusion, poor medication and follow up visits adherence were correlated with unfavorable cardiovascular risk factors control. Rural health care services can improve adherence to follow up visits in patients with non communicable disease.

## Ethical Approval

Design and protocol of the study approved and funded by Vice-Chancellor for Research of Guilan University of Medical Sciences and International Cardiovascular Research Center (ICRC) (ethical code No. IR.GUMS.REC.1394.407).

## Conflict of Interests

The authors declare no conflict of interests.

## Acknowledgments

This study was funded by research chancellor of Guilan University of Medical Sciences. The authors’ heartfelt thanks are extended to all the patients who so graciously agreed to participate in this study and come from far way to study location. The authors thank all staff of Guilan Interventional Cardiovascular Research Center for their support throughout the project.
